# Examining adherence to oral anticancer medications through a human factors engineering framework: Protocol for a scoping review

**DOI:** 10.1371/journal.pone.0274963

**Published:** 2022-09-22

**Authors:** Irene D. Lin, Matthew B. Shotts, Ahmad Al-Hader, Kellie Jones Weddle, Richard J. Holden, Emily L. Mueller, Monica R. Macik, Mirian Ramirez, Ephrem Abebe

**Affiliations:** 1 Department of Pharmacy Practice, Purdue University College of Pharmacy, West Lafayette, Indiana, United States of America; 2 Department of Medicine, School of Medicine, Indiana University, Indianapolis, Indiana, United States of America; 3 Department of Health and Wellness Design, School of Public Health-Bloomington, Indiana University, Bloomington, Indiana, United States of America; 4 Regenstrief Institute, Inc, Indianapolis, Indiana, United States of America; 5 Department of Pediatrics, School of Medicine, Indiana University, Indianapolis, Indiana, United States of America; 6 Department of Pharmacy, Eskenazi Health, Indianapolis, Indiana, United States of America; 7 Ruth Lilly Medical Library, School of Medicine, Indiana University, Indianapolis, Indiana, United States of America; 8 Regenstrief Center for Healthcare Engineering, Purdue University, West Lafayette, Indiana, United States of America; UCSI University, MALAYSIA

## Abstract

**Background:**

The number of oral anticancer medications has increased over the past few decades, opening new possibilities in cancer care and improving convenience for patients and caregivers. However, adherence levels continue to be suboptimal, potentially jeopardizing therapeutic benefits. Poor adherence levels may indicate gaps in current strategies and interventions aimed at enhancing medication adherence and the extent to which they address the complex and multi-faceted medication management needs of patients and their caregivers. Beyond commonly understood barriers (e.g., forgetting to take medications), adherence interventions must address systemic barriers that may not be fully appreciated by members of the healthcare system. This scoping review aims to apply a systems framework (human factors engineering framework) to examine system elements targeted by adherence enhancing interventions.

**Methods:**

Studies published in English, reporting adherence interventions for oral anticancer medications with adherence and/or persistence as primary outcome measures will be included in this review. We will search the following electronic databases with no limits on dates: Ovid MEDLINE, Cochrane Library, Web of Science Core Collection, Embase, CINAHL Complete, PsycInfo, and Scopus. Two reviewers will independently screen study titles and abstracts for inclusion with a third reviewer adjudicating conflicts. Full text of included articles will be used to extract information on systemic barriers targeted by adherence interventions as well as information about intervention type, outcomes, and study characteristics. Extracted information will be synthesized to generate a summary of work system factors targeted by adherence interventions.

**Discussion:**

Through application of a systems-based approach, this scoping review is expected to shed light on the complex and multifaceted nature of factors influencing adherence to oral anticancer agents. The review may also identify areas that are ripe for further research.

## Introduction

Over the past two decades, oncology treatment development has become the largest growing therapeutic area globally [1, 2]. In 2020, more than 1,300 drugs under development worldwide were designated for oncology treatment. This high level of pipeline activity introduced new cancer treatments and expanded oncology therapeutic options [2, 3]. A key category within this growing list, oral anticancer medications, have become the standard of care across several types of cancer, including metastatic melanoma, leukemia, non–small-cell lung cancer, and renal cell carcinoma [4]. In 2018, oral anticancer agents comprised more than 50% of the newly approved anticancer agents, demonstrating the thriving role of these agents in cancer treatment [2, 4].

The shift towards orally administered oncology therapy instead of the traditional intravenous administration has, in part, been driven by availability of highly effective targeted agents, perceived benefit in terms of convenience, and improved quality of life [5, 6]. These advantages have expanded the use of oral anticancer agents in both curative and palliative settings and have contributed to improved patient survival [6, 7]. Despite these benefits, treatment nonadherence remains a major barrier to realizing the full therapeutic potential of oral anticancer medications. Rates of adherence to these agents were reported to be as low as 30 to 46 percent [8, 9].

A growing body of literature has reported interventions designed to enhance adherence to oral anticancer agents [7, 10–12]. A recent scoping review [7] reported that interventions designed to improve adherence to oral anticancer medications predominantly employed education and counseling about the safe and appropriate use of these medications [7]. This review primarily focused on a broad description of adherence interventions. Given the complex and multifaceted factors influencing adherence, interventions should address the multiple needs of patients managing their medication regimens while also being sensitive to the social, cultural, technological, and organizational contexts within which oral anticancer medications are administered and managed [13]. A recent push towards multilevel interventions [14], including by the U.S National Cancer Institute [15], acknowledges the importance of looking beyond singular factors that influence health behaviors, such as medication adherence. For example, when studying medication adherence, instead of narrowly focusing only on individual behaviors (e.g., whether a patient forgets to take oral anticancer medication), a more systematic approach would examine: the patient behavior (e.g., taking or forgetting a medication); the tasks required of patients to maintain optimal adherence (e.g., learning about medications, filling prescriptions, keeping track of administration time, etc.); the tools or technologies to support medication use (e.g., organizing, tracking, etc.); the cultural, social, and physical context within which the medication is being used (e.g., at home, while traveling, in the presence of other individuals, etc.) Given this, examining interventions through a systems lens is critical.

**Fig 1:**
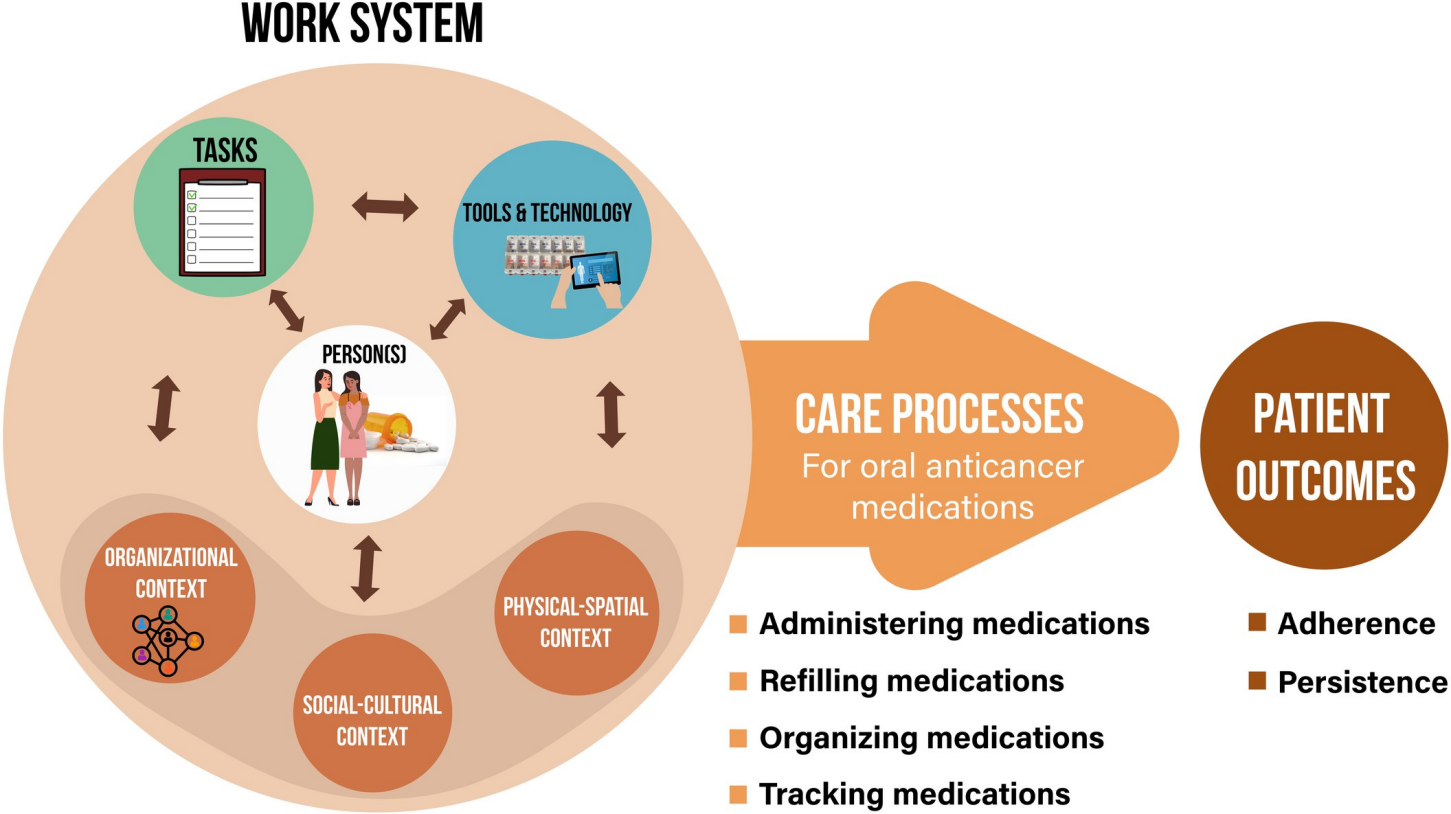
Adaptation of the patient work system model [20] for oral anticancer medication use.

The scientific discipline of Human Factors Engineering (HFE) offers system-oriented frameworks to examine factors that influence adherence to oral anticancer medications. One family of frameworks called the Systems Engineering Initiative for Patient Safety (SEIPS) models [16–18] draw upon the well-known Structure-Process-Outcome model of care quality originally developed by Donabedian [19]. The SEIPS family of models expand the “Structure” component into a “Work System” comprised of multiple interacting elements (e.g., tools and technology, tasks, organizational context, work environment) that influence processes and outcomes of care. An adaptation of the SEIPS model to the patient context, known as the Patient Work System [20], describes the following work system components (Fig 1): 1) person(s) and their attributes (e.g., a patient diagnosed with cancer, caregivers supporting the patient); 2) tasks and their attributes (e.g., patient administering, organizing, and keeping track of oral anticancer medication; difficulty/complexity of tasks); 3) tools and technologies (e.g., pill organizer, medication reminder, used by the patient); and 4) contextual factors (including organizational, socio-cultural, and physical-spatial contexts).

The SEIPS family of models and its adaptations have been widely used to study safety and performance of clinicians and patients in multiple healthcare settings [20–22]. Application of a HFE framework such as the patient work system to the problem of adherence to oral anticancer medications offers a systems lens through which interventions, and their impact on individual work system elements, can be examined. Given the multifaceted and complex nature of work involved when managing oral anticancer medications (and contributing role to adherence or nonadherence), it is critical to employ frameworks that enable understanding of medication adherence for its complexity. The goal of this scoping review is thus to 1) apply the patient work system model and characterize how adherence to oral anticancer medications have been conceptualized; and 2) examine work system elements addressed by interventions designed to enhance adherence to oral anticancer agents.

## Methods

### Protocol registration

This scoping review is being conducted following the reporting guidance from the Preferred Reporting Items for Systematic Reviews and Meta-Analyses extension for Scoping Reviews (PRISMA-ScR) statement [23]. The study protocol is pending registration with the International Prospective Register of Systematic Reviews (PROSPERO) and will be updated when a registration number becomes available.

### Study eligibility

#### Inclusion criteria

We will include studies published in English reporting interventions aimed at enhancing adherence to oral anticancer medications, with adherence and/or persistence as primary outcome measures. Any type of adherence-enhancing intervention will be considered, including, but not limited to, behavioral interventions (e.g., daily treatment diaries, utilizing weekly pillboxes, adopting cognitive behavioral therapy), technology use (e.g., mobile apps, text message reminders, patient report portal), and social context interventions (e.g., home care visits, social care, psychosocial support) [24–30]. Quantitative, qualitative, and mixed-method study designs will be considered for inclusion. Studies conducted in any country and patient age group will be evaluated for inclusion.

#### Exclusion criteria

Studies that do not report adherence intervention aimed at oral anticancer agents and adherence and/or persistence as primary outcome measures will be excluded. Studies reporting patient use of oral anticancer agents for non-oncological conditions or off-label use will be excluded. Additional exclusion criteria include oral anticancer agent use in clinical trial settings and interventions delivered in institutionalized settings (e.g., nursing care facilities).

### Outcome measures

The outcomes of interest will be adherence or persistence to oral anticancer agents, as measured by direct pill counts, patient self-reports, electronic monitoring systems, biological assays, or other standard methods (e.g., calculated from pharmacy administrative/claims data) [26, 31].

### Search strategy

A search of articles has been completed with no start date specified using the following seven databases: Ovid MEDLINE(R), Cochrane Library, Web of Science Core Collection, Embase, CINAHL Complete, PsycInfo, and Scopus. The search consisted of keywords and controlled vocabulary used in the title and the abstract as free-text words. Terms associated with the concepts ‘medication adherence’ and ‘oral administration’ were combined with terms related to ‘cancer.’ We included proximity or adjacency operators (NEAR or ADJ) to connect search terms, which were also disaggregated using truncation and wildcard symbols, allowed in most databases, to capture different words endings. Limits were added to the searches in order to exclude non-English articles. Prior to commencing synthesis, a supplementary search will be conducted to include additional articles that may have been published since the initial search. The supplementary search will also include key words related to broad classes of anticancer medications to ensure inclusion of articles only referencing drug class names. The results from all databases will be imported into EndNote® and saved without deduplication. Duplicated references will be identified and removed in COVIDENCE® prior to further screening. The search strategy is available in S1 Appendix: Literature search. The search strategy was developed by the research team in consultation with a medical librarian (MR), who was responsible for leading and executing the search.

### Data selection and screening process

Screening articles for relevance will be conducted after all retrieved articles have been imported into an online database called COVIDENCE®. COVIDENCE® is a proprietary, purpose-built tool that facilitates screening by presenting individual study title and abstracts alongside three options: “Yes”, “No”, and “Maybe”. A reviewer can tap “Yes” if study meets eligibility criteria, “No” to exclude the study, or “Maybe” if unsure about the study. Two reviewers (IDL and MBS) will independently review and screen study title and abstracts for inclusion, with a third reviewer (EA) adjudicating conflicts between the two reviewers. All three reviewers will regularly meet to discuss the screening process and adjudicate any outstanding conflicts through consensus agreement. Following this initial screening, full text articles will be retrieved for retained studies and further reviewed to ensure they meet inclusion criteria. Reference lists of included studies will also be reviewed to identify additional articles that may be relevant for inclusion.

### Assessment of quality

All studies will be reviewed carefully throughout the screening process prior to data extraction. Included articles must be peer-reviewed to ensure high quality. Given the nature of this review, a formal bias assessment is not applicable.

### Data extraction

Full text of the final selected articles will be imported into a reference management software (EndNote ®). The study team will then extract information on work system elements using a structured data collection tool based on the patient work system framework (Fig 1). An excel spreadsheet formatted using the patient work system framework has been developed and pilot tested by our research team. Specifically, information on adherence intervention features in relation to the following work system components will be collected: person(s) involved, tasks, tools and technology, physical-spatial context, social-cultural context, and organizational context [20]. We will also extract information on how adherence measures have been characterized and outcomes reported in individual studies. Additional data extraction will include information on study characteristics, including author information, publication year, geographic location, study design, characteristics of the patient population, study context, the types and descriptions of adherence-enhancing intervention, adherence assessment methods, and core study findings. Two trained study team members (IDL and MBS) will independently extract information from individual articles. Results will be reconciled in the presence of a third reviewer (EA). Disagreements will be addressed through discussion and consensus.

### Data synthesis and presentation

Final data will be reviewed by the three primary reviewers and practicing oncology team members (two hematologist/oncologists (pediatric and adult) and two oncology pharmacists). The excel spreadsheet containing relevant data elements will be presented to the entire team and discussed to develop consensus on work system elements addressed by adherence interventions. The data will then be collated and categorized under the work system elements of the patient work system framework (Table 1). Studies will be characterized based on the focus of reported interventions and this will be presented using a table grid or another graphical method that depicts how studies and their interventions fit within the human factors engineering framework. Findings will be reported consistent with PRISMA-ScR reporting guidelines [23].

**Table 1:** The patient work system study’s framework.

Work System Elements
Persons (E.g., patients, healthcare providers, and informal caregivers)
Tasks (E.g., characteristics of care tasks such as difficulty, complexity, variety, ambiguity, conflict and inertia, and sequence
Tools and Technology (E.g., usability, accessibility, familiarity, level of automation, portability, and functionality)
Physical-Spatial Context (E.g., distances and spaces, working surfaces, physical workspace, light/noise/vibration)
Social-Cultural Context (E.g., social influence, norms, social support, social engagement, and culture)
Organizational Context (E.g., work coordination, communication, policies, routines, workload demands, finances, and facility services)

## Discussion

The number of oral anticancer medications joining the cancer therapeutic toolkit will continue to grow, with several candidates already in the drug development pipeline. While this is an advantage for patients and the oncology care community, this also means a greater portion of care and medication management tasks are being shifted onto patients and their informal caregivers at home and in the community.

The persistent and worrying levels of poor adherence associated with long term use of oral anticancer medications may point to gaps in current approaches and strategies towards adherence improvement. Through application of a systems-based approach (i.e., a HFE framework), this scoping review is expected to shed light on the complex and multifaceted nature of factors influencing adherence to oral anticancer agents. The review may also identify areas that are ripe for further research. Additional details on the proposed scoping review, including findings from included articles and their implications as well as methodological contributions and limitations will be discussed in a planned future manuscript.

## Supporting information

S1 Appendix (DOCX)

S2 Appendix Preferred Reporting Items for Systematic reviews and Meta-Analyses extension for Scoping Reviews (PRISMA-ScR) checklist. (DOCX)
