# Supplementary material for: Examining adherence to oral anticancer medications through a human factors engineering framework: Protocol for a scoping review
Source: PLoS One. 2022 Sep 22;17(9):e0274963. doi: 10.1371/journal.pone.0274963 (PMC9499223; doi:10.1371/journal.pone.0274963)
Supplement: S1 Appendix — (DOCX) [file pone.0274963.s001.docx]

Appendix A

**Literature searches**

Ovid MEDLINE(R)
Search: December 23, 2020

| ID | Search history | Results |
| --- | --- | --- |
| 1 | "medication adherence".sh. | 19988 |
| 2 | "patient compliance".sh. | 58278 |
| 3 | "treatment adherence and compliance".sh. | 598 |
| 4 | 4 ((adhere* or adhering or nonadher* or overadher* or persist* or nonpersit* or overpersist* or compliant or compliance or comply* or complies or complied or noncomplian* or overcomplian*) adj11 (agent* or chemotherap* or drug* or endocrin* or formula* or hormon* or inhibitor* or medicament* or medication* or medicine* or patient* or therap* or treatment*)).ab,kw,ti. | 249335 |
| 5 | 1 or 2 or 3 or 4 | 290588 |
| 6 | exp "antineoplastic agents"/ | 1114207 |
| 7 | "anticarcinogenic agents".sh. | 10190 |
| 8 | exp neoplasms/ | 3394589 |
| 9 | (antineoplastic* or "anti-neoplastic*" or anticancer or chemotherap* cytostatic* or anticarcinogenic or "anti-carcinogenic" or antitumo?r* or cytotoxic* or oncolytic* or neoplasm* or cancer* or tum?r* or oncolog* or malignan* or carcinoma* or metastas* or metastatic* or sarcoma* or melanoma* or microcarcinoma* or leukemia* or lymphoma* or myeloma* or myelodysplas*).ab,kw,ti. | 3788903 |
| 10 | 6 or 7 or 8 or 9 | 5067005 |
| 11 | exp "administration, oral"/ | 148035 |
| 12 | mouth.sh. | 22180 |
| 13 | (oral or orally or buccal or mouth or sublingual or capsule* or pill* or tablet* or suspension or "liquid dosage" or "liquid formula*" or "liquid intake" or "liquid preparation*" or "liquid medic*").ab,kw,ti. | 964594 |
| 14 | 11 or 12 or 13 | 1008725 |
| 15 | 5 and 10 and 14 | 4871 |
| 16 | limit 15 to english language | 4517 |

Cochrane Library
Search: December 23, 2020

| ID | Search history | Results |
| --- | --- | --- |
| #1 | MeSH descriptor: [Medication Adherence] this term only | 2281 |
| #2 | MeSH descriptor: [Patient Compliance] this term only | 9726 |
| #3 | MeSH descriptor: [Treatment Adherence and Compliance] this term only | 79 |
| #4 | ((adhere* or adhering or nonadher* or overadher* or persist* or nonpersit* or overpersist* or compliant or compliance or comply* or complies or complied or noncomplian* or overcomplian*) NEAR/11 (agent* or chemotherap* or drug* or endocrin* or formula* or hormon* or inhibitor* or medicament* or medication* or medicine* or patient* or therap* or treatment*)):ti,ab,kw | 69539 |
| #5 | #1 or #2 or #3 or #4 | 69539 |
| #6 | MeSH descriptor: [Antineoplastic Agents] explode all trees | 12190 |
| #7 | MeSH descriptor: [Anticarcinogenic Agents] this term only | 280 |
| #8 | MeSH descriptor: [Neoplasms] explode all trees | 79636 |
| #9 | (antineoplastic* or "anti-neoplastic*" or anticancer or chemotherap* cytostatic* or anticarcinogenic or "anti-carcinogenic" or antitumo* or cytotoxic* or oncolytic* or neoplasm* or cancer* or tumor or tumour* or oncolog* or malignan* or carcinoma* or metastas* or metastatic* or sarcoma* or melanoma* or microcarcinoma* or leukemia* or lymphoma* or myeloma* or myelodysplas*):ti,ab,kw | 236235 |
| #10 | #6 or #7 or #8 or #9 | 241000 |
| #11 | MeSH descriptor: [Administration, Oral] explode all trees | 24057 |
| #12 | MeSH descriptor: [Mouth] this term only | 572 |
| #13 | (oral or orally or buccal or mouth or sublingual or capsule* or pill* or tablet* or suspension or "liquid dosage" or "liquid formula*" or "liquid intake" or "liquid preparation*" or "liquid medic*"):ti,ab,kw 249554 |  |
| #14 | #11 or #12 or #13 | 249554 |
| #15 | #5 and #10 and #14 | 2294 |

Web of Science Core Collection
Search: December 23, 2020

| ID | Search history | Results |
| --- | --- | --- |
| # 4 | #3 AND #2 AND #1 *AND* LANGUAGE: (English)  Indexes=SCI-EXPANDED, SSCI, A&HCI, CPCI-S, CPCI-SSH, BKCI-S, BKCI-SSH, ESCI Timespan=1985-2020 | 3,525 |
| # 3 | TS= (oral or orally or buccal or mouth or sublingual or capsule* or pill* or tablet* or suspension or "liquid dosage" or "liquid formula*" or "liquid intake" or "liquid preparation*" or "liquid medic*")  Indexes=SCI-EXPANDED, SSCI, A&HCI, CPCI-S, CPCI-SSH, BKCI-S, BKCI-SSH, ESCI Timespan=1985-2020 | 1,201,107 |
| # 2 | TS=(antineoplastic* or "anti-neoplastic*" or anticancer or chemotherap* cytostatic* or anticarcinogenic or "anti-carcinogenic" or antitumo$r* or cytotoxic* or oncolytic* or neoplasm* or cancer* or tum$r* or oncolog* or malignan* or carcinoma* or metastas* or metastatic* or sarcoma* or melanoma* or microcarcinoma* or leukemia* or lymphoma* or myeloma* or myelodysplas*)  Indexes=SCI-EXPANDED, SSCI, A&HCI, CPCI-S, CPCI-SSH, BKCI-S, BKCI-SSH, ESCI Timespan=1985-2020 | 4,432,815 |
| # 1 | TS=((adhere* or adhering or nonadher* or overadher* or persist* or nonpersit* or overpersist* or compliant or compliance or comply* or complies or complied or noncomplian* or overcomplian*) NEAR/10 (agent* or chemotherap* or drug* or endocrin* or formula* or hormon* or inhibitor* or medicament* or medication* or medicine* or patient* or therap* or treatment*) )  Indexes=SCI-EXPANDED, SSCI, A&HCI, CPCI-S, CPCI-SSH, BKCI-S, BKCI-SSH, ESCI Timespan=1985-2020 | 232,598 |

EMBASE
Search: December 23, 2020

| ID | Search history | Results |
| --- | --- | --- |
| #17 | #15 AND #16 | 9,904 |
| #16 | english:la | 32,377,475 |
| #15 | #6 AND #10 AND #14 | 10,432 |
| #14 | #11 OR #12 OR #13 | 1,649,122 |
| #13 | oral:ti,ab,kw OR orally:ti,ab,kw OR buccal:ti,ab,kw OR mouth:ti,ab,kw OR sublingual:ti,ab,kw OR capsule*:ti,ab,kw OR pill*:ti,ab,kw OR tablet*:ti,ab,kw OR suspension:ti,ab,kw OR 'liquid dosage':ti,ab,kw OR 'liquid formula*':ti,ab,kw OR 'liquid intake':ti,ab,kw OR 'liquid preparation*':ti,ab,kw OR 'liquid medic*':ti,ab,kw | 1,352,451 |
| #12 | 'mouth'/de | 49,631 |
| #11 | 'oral drug administration'/de | 400,851 |
| #10 | #7 OR #8 OR #9 | 6,575,189 |
| #9 | antineoplastic*:ti,ab,kw,de OR 'anti-neoplastic*':ti,ab,kw,de OR anticancer:ti,ab,kw,de OR chemotherap*:ti,ab,kw,de OR cytostatic*:ti,ab,kw,de OR anticarcinogenic:ti,ab,kw,de OR 'anti-carcinogenic':ti,ab,kw,de OR antitumor*:ti,ab,kw,de OR cytotoxic*:ti,ab,kw,de OR oncolytic*:ti,ab,kw,de OR neoplasm*:ti,ab,kw,de OR cancer*:ti,ab,kw,de OR tumor*:ti,ab,kw,de OR tumour*:ti,ab,kw,de OR oncolog*:ti,ab,kw,de OR malignan*:ti,ab,kw,de OR carcinoma*:ti,ab,kw,de OR metastas*:ti,ab,kw,de OR metastatic*:ti,ab,kw,de OR sarcoma*:ti,ab,kw,de OR melanoma*:ti,ab,kw,de OR microcarcinoma*:ti,ab,kw,de OR leukemia*:ti,ab,kw,de OR lymphoma*:ti,ab,kw,de OR myeloma*:ti,ab,kw,de OR myelodysplas*:ti,ab,kw,de | 6,575,189 |
| #8 | 'neoplasm'/de | 565,143 |
| #7 | 'antineoplastic agent'/de | 313,426 |
| #6 | #1 OR #2 OR #3 OR #4 OR #5 | 473,110 |
| #5 | ((adhere* OR adhering OR nonadher* OR overadher* OR persist* OR nonpersit* OR overpersist* OR compliant OR compliance OR comply* OR complies OR complied OR noncomplian* OR overcomplian*) NEAR/10 (agent* OR chemotherap* OR drug* OR endocrin* OR formula* OR hormon* OR inhibitor* OR medicament* OR medication* OR medicine* OR patient* OR therap* OR treatment*)):ti,ab,kw | 390,049 |
| #4 | 'medication adherence monitoring system'/de | 222 |
| #3 | 'medication compliance'/de | 33,662 |
| #2 | 'patient compliance'/de | 134,726 |
| #1 | 'adherence'/de | 61 |

CINAHL Complete (EBSCOhost Interface)
Search: December 23, 2020

| ID | Search history | Results |
| --- | --- | --- |
| S20 | ( S6 AND S12 AND S18 ) AND LA English | 1,497 |
| S19 | (S6 AND S12 AND S18) | 1,531 |
| S18 | S13 OR S14 OR S15 OR S16 OR S17 | 226,715 |
| S17 | SU (oral or orally or buccal or mouth or sublingual or capsule* or pill* or tablet* or suspension or "liquid dosage" or "liquid formula*" or "liquid intake" or "liquid preparation*" or "liquid medic*") | 80,523 |
| S16 | AB oral or orally or buccal or mouth or sublingual or capsule* or pill* or tablet* or suspension or "liquid dosage" or "liquid formula*" or "liquid intake" or "liquid preparation*" or "liquid medic*" | 154,613 |
| S15 | TI (oral or orally or buccal or mouth or sublingual or capsule* or pill* or tablet* or suspension or "liquid dosage" or "liquid formula*" or "liquid intake" or "liquid preparation*" or "liquid medic*") | 73,067 |
| S14 | MH mouth+ | 18,352 |
| S13 | MH administration, oral+ | 23,737 |
| S12 | S7 OR S8 or S9 OR S10 OR S11 | 842,082 |
| S11 | SU (antineoplastic* or "anti-neoplastic*" or anticancer or chemotherap* cytostatic* or anticarcinogenic or "anti-carcinogenic" or antitumo#r* or cytotoxic* or oncolytic* or neoplasm* or cancer* or tumo#r* or oncolog* or malignan* or carcinoma* or metastas* or metastatic* or sarcoma* or melanoma* or microcarcinoma* or leukemia* or lymphoma* or myeloma* or myelodysplas*) | 614,673 |
| S10 | AB (antineoplastic* or "anti-neoplastic*" or anticancer or chemotherap* cytostatic* or anticarcinogenic or "anti-carcinogenic" or antitumo#r* or cytotoxic* or oncolytic* or neoplasm* or cancer* or tumo#r* or oncolog* or malignan* or carcinoma* or metastas* or metastatic* or sarcoma* or melanoma* or microcarcinoma* or leukemia* or lymphoma* or myeloma* or myelodysplas*) | 436,613 |
| S9 | TI (antineoplastic* or "anti-neoplastic*" or anticancer or chemotherap* cytostatic* or anticarcinogenic or "anti-carcinogenic" or antitumo#r* or cytotoxic* or oncolytic* or neoplasm* or cancer* or tumo#r* or oncolog* or malignan* or carcinoma* or metastas* or metastatic* or sarcoma* or melanoma* or microcarcinoma* or leukemia* or lymphoma* or myeloma* or myelodysplas*) | 478,974 |
| S8 | MH neoplasms+ | 565,327 |
| S7 | MH Antineoplastic Agents+ | 120,156 |
| S6 | (S1 OR S2 OR S3 OR S4 OR S5) | 105,380 |
| S5 | SU ((adhere* or adhering or nonadher* or overadher* or persist* or nonpersit* or overpersist* or compliant or compliance or comply* or complies or complied or noncomplian* or overcomplian*) N10 (agent* or chemotherap* or drug* or endocrin* or formula* or hormon* or inhibitor* or medicament* or medication* or medicine* or patient* or therap* or treatment*)) | 52,091 |
| S4 | AB ((adhere* or adhering or nonadher* or overadher* or persist* or nonpersit* or overpersist* or compliant or compliance or comply* or complies or complied or noncomplian* or overcomplian*) N10 (agent* or chemotherap* or drug* or endocrin* or formula* or hormon* or inhibitor* or medicament* or medication* or medicine* or patient* or therap* or treatment*)) | 66,479 |
| S3 | TI ((adhere* or adhering or nonadher* or overadher* or persist* or nonpersit* or overpersist* or compliant or compliance or comply* or complies or complied or noncomplian* or overcomplian*) N10 (agent* or chemotherap* or drug* or endocrin* or formula* or hormon* or inhibitor* or medicament* or medication* or medicine* or patient* or therap* or treatment*)) | 17,903 |
| S2 | MH Medication Compliance+ | 20,794 |
| S1 | MH Patient Compliance+ | 51,730 |

PsycINFO 1887-current (EBSCOhost Interface)
Search: December 23, 2020

| ID | Search history | Results |
| --- | --- | --- |
| S23 | (LA English) AND (S21 AND S22) | 158 |
| S22 | LA English | 4,611,625 |
| S21 | S8 AND S15 AND S20 | 160 |
| S20 | S16 OR S17 OR S18 OR S19 | 92,371 |
| S19 | SU oral or orally or buccal or mouth or sublingual or capsule* or pill* or tablet* or suspension or "liquid dosage" or "liquid formula*" or "liquid intake" or "liquid preparation*" or "liquid medic*" | 38,919 |
| S18 | AB oral or orally or buccal or mouth or sublingual or capsule* or pill* or tablet* or suspension or "liquid dosage" or "liquid formula*" or "liquid intake" or "liquid preparation*" or "liquid medic*" | 69,812 |
| S17 | TI oral or orally or buccal or mouth or sublingual or capsule* or pill* or tablet* or suspension or "liquid dosage" or "liquid formula*" or "liquid intake" or "liquid preparation*" or "liquid medic*" | 13,715 |
| S16 | SU "administration, oral" | 3,741 |
| S15 | S9 OR S10 OR S11 OR S12 OR S13 OR S14 | 96,391 |
| S14 | SU antineoplastic* or "anti-neoplastic*" or anticancer or chemotherap* cytostatic* or anticarcinogenic or "anti-carcinogenic" or antitumo#r* or cytotoxic* or oncolytic* or neoplasm* or cancer* or tumo#r* or oncolog* or malignan* or carcinoma* or metastas* or metastatic* or sarcoma* or melanoma* or microcarcinoma* or leukemia* or lymphoma* or myeloma* or myelodysplas* | 73,234 |
| S13 | AB antineoplastic* or "anti-neoplastic*" or anticancer or chemotherap* cytostatic* or anticarcinogenic or "anti-carcinogenic" or antitumo#r* or cytotoxic* or oncolytic* or neoplasm* or cancer* or tumo#r* or oncolog* or malignan* or carcinoma* or metastas* or metastatic* or sarcoma* or melanoma* or microcarcinoma* or leukemia* or lymphoma* or myeloma* or myelodysplas* | 84,856 |
| S12 | TI antineoplastic* or "anti-neoplastic*" or anticancer or chemotherap* cytostatic* or anticarcinogenic or "anti-carcinogenic" or antitumo#r* or cytotoxic* or oncolytic* or neoplasm* or cancer* or tumo#r* or oncolog* or malignan* or carcinoma* or metastas* or metastatic* or sarcoma* or melanoma* or microcarcinoma* or leukemia* or lymphoma* or myeloma* or myelodysplas* | 47,349 |
| S11 | SU "Antineoplastic Drugs" | 224 |
| S10 | SU "anticarcinogenic agents" | 31 |
| S9 | SU "antineoplastic agents" | 2,108 |
| S8 | S1 OR S2 OR S3 OR S4 OR S5 OR S6 OR S7 | 51,916 |
| S7 | SU (adhere* or adhering or nonadher* or overadher* or persist* or nonpersit* or overpersist* or compliant or compliance or comply* or complies or complied or noncomplian* or overcomplian*) N10 (agent* or chemotherap* or drug* or endocrin* or formula* or hormon* or inhibitor* or medicament* or medication* or medicine* or patient* or therap* or treatment*) | 24,989 |
| S6 | AB (adhere* or adhering or nonadher* or overadher* or persist* or nonpersit* or overpersist* or compliant or compliance or comply* or complies or complied or noncomplian* or overcomplian*) N10 (agent* or chemotherap* or drug* or endocrin* or formula* or hormon* or inhibitor* or medicament* or medication* or medicine* or patient* or therap* or treatment*) | 39,319 |
| S5 | TI (adhere* or adhering or nonadher* or overadher* or persist* or nonpersit* or overpersist* or compliant or compliance or comply* or complies or complied or noncomplian* or overcomplian*) N10 (agent* or chemotherap* or drug* or endocrin* or formula* or hormon* or inhibitor* or medicament* or medication* or medicine* or patient* or therap* or treatment*) | 8,198 |
| S4 | SU "Treatment Compliance" | 15,697 |
| S3 | SU "treatment adherence and compliance" | 77 |
| S2 | SU "patient compliance" | 10,864 |
| S1 | SU "medication adherence" | 4,681 |

Scopus
Search: December 23, 2020

( ( TITLE-ABS-KEY ( ( *adhere* OR adhering OR *persist* OR compliant OR compliance OR comply* OR complies OR complied OR noncomplian* OR overcomplian* ) W/10 ( agent* OR chemotherap* OR drug* OR endocrin* OR formula* OR hormon* OR inhibitor* OR medicament* OR medication* OR medicine* OR patient* OR therap* OR treatment* ) ) ) AND ( TITLE-ABS-KEY ( *neoplastic* OR chemotherap* AND cytostatic* OR *carcinogenic OR cytotoxic* OR oncolytic* OR neoplasm* OR *cancer* OR *tumor* OR *tumour* OR oncolog* OR malignan* OR carcinoma* OR metastas* OR metastatic* OR sarcoma* OR melanoma* OR microcarcinoma* OR leukemia* OR lymphoma* OR myeloma* OR myelodysplas* ) ) AND ( TITLE-ABS-KEY ( oral OR orally OR buccal OR mouth OR sublingual OR capsule* OR pill* OR tablet* OR suspension OR "liquid dosage" OR "liquid formula*" OR "liquid intake" OR "liquid preparation*" OR "liquid medic*" ) ) ) AND ( LANGUAGE ( english ) )

2,900 document results
