# Supplementary material for: Examining adherence to oral anticancer medications through a human factors engineering framework: Protocol for a scoping review
Source: PLoS One. 2022 Sep 22;17(9):e0274963. doi: 10.1371/journal.pone.0274963 (PMC9499223; doi:10.1371/journal.pone.0274963)
Supplement: S2 Appendix — (DOCX) [file pone.0274963.s002.docx]

**Appendix B: Preferred Reporting Items for Systematic reviews and Meta-Analyses extension for Scoping Reviews (PRISMA-ScR) Checklist**

| **SECTION** | **ITEM** | **PRISMA-ScR CHECKLIST ITEM** | **REPORTED ON PAGE #** |
| --- | --- | --- | --- |
| **TITLE** | | | |
| Title | 1 | Identify the report as a scoping review. | Title Page |
| **ABSTRACT** | | | |
| Structured summary | 2 | Structured abstract provided | 2 |
| **INTRODUCTION** | | | |
| Rationale | 3 | This review applies a systems engineering lens (work system model) to identify contextual factors influenced by adherence interventions directed at orally administered anticancer agents. This will help identify current gaps in adherence interventions and inform future work and intervention design to target the multifaceted nature of medication adherence. | 3-4 |
| Objectives | 4 | Applying work system model, what work system elements are addressed by interventions designed to enhance adherence to oral anticancer agents | 4 |
| **METHODS** | | | |
| Protocol and registration | 5 | This submission is the protocol for planned scoping review. Registration request submitted to PROSPERO and pending registration number. | 6 |
| Eligibility criteria | 6 | We plan to include all empiric studies published in English and reporting interventions aimed at enhancing adherence to oral anticancer medications. Qualitative, quantitative, and mixed-design studies are considered for inclusion. There will be no restrictions in geographic region and age of study participants. There is also no limit on start date for publications. Articles until Dec 2021 will be included for this study. Supplemental search will be conducted later to include articles that may have been published since Dec 2021. | 6 |
| Information sources* | 7 | The following databases are being used: Ovid MEDLINE(R), Cochrane Library, Web of Science Core Collection, Embase, CINAHL Complete, PsycInfo, and Scopus. | 7 |
| Search | 8 | Search strategy provided in Appendix | Appendix A |
| Selection of sources of evidence† | 9 | Two members of the team will independently screen titles, abstracts, full text articles for inclusion. Any conflicts will be resolved in the presence of a third reviewer. | 7 |
| Data charting process‡ | 10 | A calibrated form as been developed by the team and tested. | 8 |
| Data items | 11 | Author  Publication year  Geographic location  Study design  Characteristics of study population  Study context  Type of adherence intervention  Adherence assessment method  Core study findings  Work system elements influence by adherence intervention | 8 |
| Critical appraisal of individual sources of evidence§ | 12 | If done, provide a rationale for conducting a critical appraisal of included sources of evidence; describe the methods used and how this information was used in any data synthesis (if appropriate). | NA |
| Synthesis of results | 13 | Data will be thematically synthesized and described to characterize features of adherence interventions and work system elements addressed by interventions | 8-9 |
| **RESULTS** | | | |
| Selection of sources of evidence | 14 | N/A | Click here to enter text. |
| Characteristics of sources of evidence | 15 | N/A | Click here to enter text. |
| Critical appraisal within sources of evidence | 16 | N/A | Click here to enter text. |
| Results of individual sources of evidence | 17 | N/A | Click here to enter text. |
| Synthesis of results | 18 | N/A | Click here to enter text. |
| **DISCUSSION** | | | |
| Summary of evidence | 19 | A summary of work system elements addressed by adherence interventions will be synthesized along with a discussion on implications for future research and considerations during adherence intervention design. | 10 |
| Limitations | 20 | Discuss the limitations of the scoping review process. | Click here to enter text. |
| Conclusions | 21 | N/A | Click here to enter text. |
| **FUNDING** | | | |
| Funding | 22 | This study does not have external funding | Click here to enter text. |

JBI = Joanna Briggs Institute; PRISMA-ScR = Preferred Reporting Items for Systematic reviews and Meta-Analyses extension for Scoping Reviews.

* Where *sources of evidence* (see second footnote) are compiled from, such as bibliographic databases, social media platforms, and Web sites.

† A more inclusive/heterogeneous term used to account for the different types of evidence or data sources (e.g., quantitative and/or qualitative research, expert opinion, and policy documents) that may be eligible in a scoping review as opposed to only studies. This is not to be confused with *information sources* (see first footnote).

‡ The frameworks by Arksey and O’Malley (6) and Levac and colleagues (7) and the JBI guidance (4, 5) refer to the process of data extraction in a scoping review as data charting*.*

§ The process of systematically examining research evidence to assess its validity, results, and relevance before using it to inform a decision. This term is used for items 12 and 19 instead of "risk of bias" (which is more applicable to systematic reviews of interventions) to include and acknowledge the various sources of evidence that may be used in a scoping review (e.g., quantitative and/or qualitative research, expert opinion, and policy document).

*From:* Tricco AC, Lillie E, Zarin W, O'Brien KK, Colquhoun H, Levac D, et al. PRISMA Extension for Scoping Reviews (PRISMAScR): Checklist and Explanation. Ann Intern Med. 2018;169:467–473. [doi: 10.7326/M18-0850](http://annals.org/aim/fullarticle/2700389/prisma-extension-scoping-reviews-prisma-scr-checklist-explanation).
